# Protected Natural Areas: In Sickness and in Health

**DOI:** 10.3390/ijerph15102182

**Published:** 2018-10-06

**Authors:** Teresa Romanillos, Roser Maneja, Diego Varga, Llorenç Badiella, Martí Boada

**Affiliations:** 1 Institute of Environmental Sciencies and Technology (ICTA), Universitat Autònoma de Barcelona (UAB), Cerdanyola del Vallés, 08193 Barcelona, Spain; tromanillos@gmail.com (T.R.); roser.maneja@uab.cat (R.M.); marti.boada@uab.cat (M.B.); 2 Hospital of St. Celoni, St. Celoni, 08470 Barcelona, Spain; 3 Geography Department, University of Girona, 17071 Girona, Spain; 4 Research Group on Statistics, Econometrics and Health (GRECS), University of Girona, 17071 Girona, Spain; 5 CIBER of Epidemiology and Public Health (CIBERESP), 28029 Madrid, Spain; 6 Servei d’Estadística Aplicada, Universitat Autònoma de Barcelona (UAB), Cerdanyola del Vallés, 08193 Barcelona, Spain; llorenc.badiella@uab.cat

**Keywords:** protected natural areas, health, disease, health-related activities

## Abstract

Numerous studies show the benefits that contact with the natural environment have for human health, but there are few studies on the role of Protected Natural Areas (PNAs), either from the preventive point of view or on their potential benefits, on individuals with health problems. A study was made of the relationship between the visitation of Montseny Natural Park and Biosphere Reserve and health, from the perspective of a population group with different diseases. A total of 250 patients resident in the areas near the park were surveyed, recording their beliefs about the benefits of nature, as well as the reasons for visiting and the activities associated with health that they carried out in the park. The pure air is the most valued benefit (27.2%), particularly for those with allergies. The majority (57%) visit the park for health reasons. High levels (82%) of exercise are recorded, especially by patients with heart diseases (85%), and 65% exercised in the park. More physical activity is mentioned among those that visit the park most often, particularly among those that carried it out for health reasons. Plants were collected for medicinal use by 39.6%. The study confirmed the significant role of the Montseny Natural Park and Biosphere Reserve as a health resource for individuals with diseases that live near it. It also corroborates the beneficial effects that the PNA provide in human health.

## 1. Introduction

There is currently a growing interest in the beneficial effects that contact with nature have for the health and well-being of humans [1,2,3].

One of the worldwide problems of public health is the growing number of non-transmissible diseases such as diabetes, obesity, cardiovascular disorders, chronic respiratory diseases, cancer, and mental disorders [4], in which it has been demonstrated that lifestyle plays an important role [5]. It has been shown that sedentarism increases the risk of suffering from diabetes, obesity, psychological disorders, and some types of cancer [6,7,8]. Furthermore, physical activity is a key element in the prevention of cardiovascular disorders and their risks [9,10].

In view of this challenge, the natural environment and green spaces can be a tool to be taken into account in public health management programs [11], given that it is established that, among other positive effects, one of the most important benefits is that of influencing lifestyle, making it possible to carry out healthy activities, such as physical exercise [12,13].

In the current scientific literature, numerous studies investigate the influence that the natural environment has on health and well-being. However, there are fewer references to the role that it plays with individuals with diseases. This fact is paradoxical, given that historically, nature has been an important therapeutic resource in the treatment of certain diseases, such as in the cases of the sanatoriums for the treatment of tuberculosis, or the therapeutic gardens in psychiatric hospitals [14,15].

The majority of studies that approach the relationship between health, and the natural environment are conducted from the perspective of urban green areas [16,17,18] and there are few investigations that study the role of protected natural areas (PNA) [19,20,21], a geographical space protected to achieve the long-term conservation of nature with associated ecosystem services and cultural values [22]. Furthermore, there are no references to health related activities and the reasons why people who live near them visit the PNA. 

This study looks at the relationship between the PNA (Montseny Natural Park–Biosphere Reserve (NP-BR) (NE, Catalonia) and health, from the perspective of a population group of individuals with diseases. The beliefs about the benefits of nature in regards to health are studied, as well as the reasons for visiting and the activities associated with health that they carry out in the Montseny NP-BR.

### 1.1. Natural Environment and Health: The Evidence

Since the first works by Ulrich in the 1980s [23], many studies have demonstrated the benefit that contact with the natural environment has on human health and well-being [24,25,26]. Overall, it is shown that there is a lower mortality [27,28,29], and greater longevity [30,31,32] among individuals that have more contact with the natural environment. Various studies have demonstrated the positive effects of this relationship, with lower rates of cardiovascular diseases [33,34,35], obesity [36,37,38] and cancer [39], as well as other health problems. There is also evidence of a lower incidence of perinatal, [40,41] as well as childhood development problems [41,42].

Contact with the natural environment also affects psychological well-being. An objective improvement in the mental health state has been demonstrated [43,44] with lower levels of depression [45,46], stress, and anxiety [43,45,47]. It has also been shown that there is a greater subjective perception of mental health [48,49,50], as well as psychological well-being [51]. Furthermore, contact with nature also brings about a subjective feeling of enjoying good health [52], with a lower number of symptoms [53], as well as a better recovery from illnesses [23,54].

Several mechanisms have been implicated as mediators of these benefits, such as air pollution filtration from vegetation [55]. On the other hand, the natural environment providing more opportunities for physical activities [56,57] and, furthermore, when exercise is performed in this environment, it is more vigorous and prolonged [11,58]. Other mediating factors are the reduction in stress levels [50,57,59], the positive feeling of contact with nature [60,61], and the perception of greater social support [52,62], given that the contact with the natural environment facilitates social interactions and cohesion, an element that contributes to health and well-being. Some studies show that the mediators have a different weight depending on gender and age [17].

The majority of studies carried out are quantitative, recording only the presence of green areas, without taking into account of what they are composed [25,27,29]. Some works have taken the size of the natural areas into consideration, with evidence that those of a larger area are more beneficial for health on being easier for physical activities [63], as well as its restorative capacity [64,65]. There is also evidence of a positive effect of the woods (forests) [66], with a possible association with reductions in stress levels [67].

In regards the qualitative nature of the natural environment, it appears that there is a positive association between the sensation of health and its perceived quality [68]. Furthermore, it has also been shown that biodiversity has a favorable influence, with evidence that the spaces with more biodiversity bring about an improved health status [69,70,71,72], as well as greater feeling of well-being [73].

One of the mediators of the benefits could be related to the influence environmental microorganisms have over the human microbiome [74]. This concept, initially expressed as a “hypothesis of hygiene” [75], in that certain immune system disorders could be associated with a lower contact with these microorganisms. A new element has currently been incorporated with “biodiversity hypothesis” [76], which considers the effects that the loss of biodiversity and less contact with natural environment may have on human health [72].

Finally, and with the interesting findings in some studies that show that the most important benefit depends on the subjective feeling of being close to nature, more than that of the objective proximity or access to these natural areas [52,57,77,78].

### 1.2. Protected Natural Areas in Relation to Health and Well-Being

Protected natural areas (PNA) deserve special attention within the natural environment, as well as their importance as conservers of biodiversity and their currently widely recognized role as providers of services associated with health and well-being [79].

Approximately 15% of the earth’s surface is conserved under the concept of a protected natural area (International Union for Conservation of Nature [IUCN]) [22]. In Europe, this figure increases to 21%, with a total of 120,000 protected areas in 52 countries [80]. At a world level, it is estimated that protected land areas receive 8 billion visits annually [81]. This important influx, converts them into privileged areas that facilitate contact with the natural environment.

In regards to health, the PNA makes different contributions, such as greater biodiversity compared to other natural areas, such as city parks [82]. Likewise, they provide psychological benefits associated with the aesthetic pleasure of the environment [83], and with a sense of connecting with nature [60,61]. This context has contributed to certain PNA having been considered as therapeutic landscapes in several cultures [84].

Besides favoring physical activity, the PNA have a higher restoring capacity on being conducive to silence and tranquility [85]. Finally, the PNA provide infrastructures, services, and information that allow specific healthy activities to be pursued, as well as those linked to the games and socializing aspects, which can also be positive for health and well-being [11,86].

But, probably, one of the most important contributions of the PNA is their contribution to human health through the ecosystems services that provide, for example, the capacity to improve the quality of the air and water by reducing contaminants [87,88]. They also play an important role in the regulation of the climate, as well as in the control of some infectious diseases [89].

In the last few decades, a growing number of programs have been developed linked to health and PNAs. Among the first projects are those of the Canadian Parks and Recreation Association (1997), and the “Healthy Parks, Healthy People” program, promoted in 1999 by those that manage the Australian protected areas (Parks Victoria). The “Healthy by Nature” was consolidated in Canada in 2006, and the First International Congress of “Healthy Parks, Healthy People” was held in Melbourne, a starting point for the National Park Service program in the U.S.A. In Europe, one of the first projects was “Natural England”, in the U.K., as well as the “Health and Protected Areas”, linked to “EUROPARC”. This program has also been developed in Spain backed by “EUROPARC-España”.

The potential that the PNA have in relation to the health and well-being of humans has historically been relatively unknown and underused [90]. This fact is paradoxical, given that the first nature parks were also created with the philosophy of their potential beneficial role for health [91].

## 2. Methodology

### 2.1. Study Area

**Figure 1:**
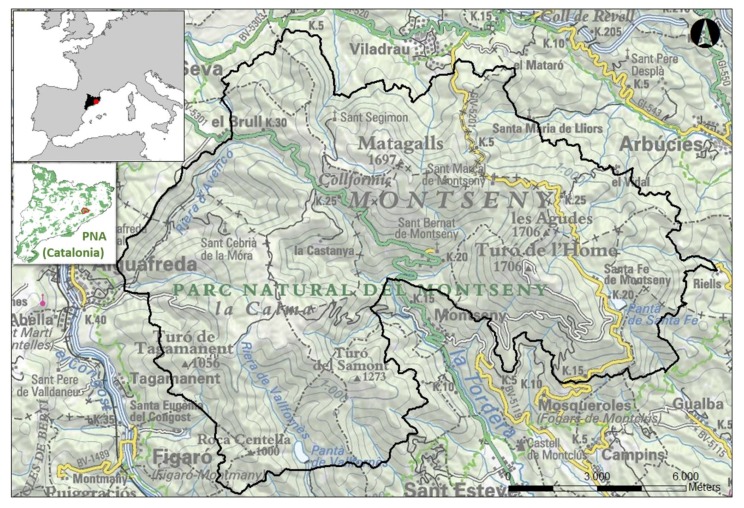
Montseny Natural Park and Biosphere Reserve.

The study is set around the Montseny Natural Park and Biosphere Reserve (NP-BR Montseny). The Montseny massif is situated in the Catalan precoastal mountain range, of which it is the most elevated and one of the highest on the non-Pyrenean Catalan mountains. It covers an area of 50,167 hectares, and is situated about 50 km from the city of Barcelona, forming the fourth metropolitan area (Figure 1). This proximity leads to an important influx, with an estimated 750,000–1,000,000 visitors annually [92].

The Montseny NP- BR has a hybrid landscape, in which nature and culture have become one of the most important natural spaces in Catalonia [93]. In the 1978, it was declared a Nature Park and in that same year, UNESCO declared it a Biosphere Reserve.

### 2.2. Participants and Data Collection

The study included an evaluation of 250 patients attending medical specialty outpatient clinics (cardiology, chest diseases, gastroenterology, neurology, and endocrinology) of the Hospital of Sant Celoni, located in Sant Celoni, a municipality near Montseny NP-BR. All the individuals of the sample lived in municipalities within the park limits or near them.

—Selection criteria: a random sample of patients attending outpatient clinics during March, and April 2016. Individuals aged between 18 and 85 years.

—Information collection procedure: a questionnaire was completed by the patients in the clinic waiting rooms. A researcher, who had nothing to do with the clinics was responsible for explaining the study, inviting participation and clarifying any doubts.

### 2.3. Questionnaire Design

The collection of information was by made using self-completed questionnaires on paper support. The questionnaire was semistructured, with dichotomic responses and multiple choices. Some questions also included an open item of free response.

Contents: An attempt was made to collect information associated with contact with the natural environment in general, and with Montseny NP-BR. The activities of the patients were recorded, highlighting those that could be related to health, as well as exercise and the collection of medicinal plants. Beliefs about nature were also evaluated in relation to health and the reasons for visiting the Montseny NP-BR. Demographic data were recorded, as well the illnesses/diseases of the patients.

### 2.4. Data Analysis

The handling of the data, which had been anonymized, fulfilled the requirements of the Clinical Research Ethics Committee of the center. An Excel file was created with the information recorded, rejecting the questionnaires with more than 20% of the questions unanswered. Open-ended responses were transcribed literally. Qualitative variables were coded, and the open questions had been were closed from the most frequent categories. A review was made of missing or anomalous data, as well as the detection of inconsistencies. No imputation process had been applied to the missing data. The responses were quantified and the results were analyzed with respect to demographic variables and in relation to the diseases. Furthermore, the beliefs, reasons, and activities have been inter-related.

Multiresponse variables were analyzed considering each category individually. The statistical analysis was performed using the R v3.1.2 software (R Foundationfor Statistical Computing, Vienna, Austria). The significance level for all the tests was set at 5% (*p* < 0.05).

Analysis of the primary variables: the possible relationship between response variables and explanatory variables was mainly examined by means of bivariate analysis. The following methods were applied:

—Quantitative variables and qualitative explanatory variables: analysis of variance, Mann–Whitney–Wilcoxon or Kruskal–Wallis test depending on the application conditions. The compliance to these has been performed using the Shapiro–Wilk and Levene tests for normality and homogeneity of variances respectively.

—Qualitative variables and qualitative explanatory variables: χ^2^-test, exact Fisher test, and likelihood ratio test, depending on the application conditions.

Correction techniques have been applied for multiple comparisons (Tukey and Bonferroni) on carrying out *a posteriori* comparisons between groups.

## 3. Results

The results are presented structured from the demographic data, the diseases, frequency of use of the park, beliefs about the benefits of nature, as well as the reasons for visiting, and health-related activities in the Montseny NP-BR. The results were analyzed in relation to the diseases of the patients.

**Figure 2:**
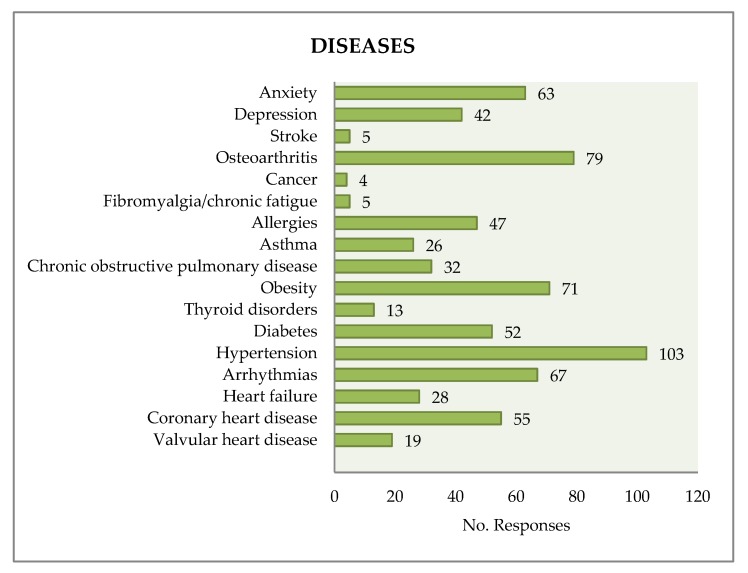
Diseases recorded in the patients.

In regards to gender, 46% of the patients were women. The mean age was 63.5 years, and 46.4% of those surveyed were over 65 years. In regards to the place of residence, the different populations are in the Montseny NP-BR area or near it. The different diseases recorded are shown in Figure 2.

### 3.1. Frequency of Visiting Montseny NP-BR

More than one-third (36.6%) of those surveyed mentioned visiting the park more than once a week. 16.7% visiting it more than once per week but less than once per month, and 46.7% visited it less than once per month. It was rarely visited by 12.6% of those that responded. In the analysis of the demographic variables, there is evidence of being visited more often in the male group.

### 3.2. Beliefs in the Benefits of Nature in Relation to Health

In regards to the specific question about whether it was considered that nature is beneficial for health, 91% of those surveyed responded positively. In regards to the reasons why it is considered that nature benefits health, after categorizing the comments from an open question, all the concepts associated with “pure air” (27.2%), followed by “tranquility” (17.6%), were highlighted as the most valued. Other reasons mentioned are those associated with “health” (10%) and the responses classified in the “exercise” (4.8%) and “spiritual” (0.8%) category. No significant differences were found in regards to age. In regards to gender, differences were found that did not reach statistical significance, with a higher evaluation of “tranquility” in the female group (Table 1).

### 3.3. Reasons for Visiting Montseny NP-BR

The most common reasons are “to enjoy” (44.8%) and “tranquility” (40.4%). It should be noted that “plant-picking“ reason was recorded in 28% of the responses. The “leisure” reason was mentioned in 16% of cases. In regards to health, 24% indicated that it was one of their reasons. However, in the specific question about whether they go to Montseny NP-BR for health reasons, 57% responded positively.

The statistical analysis by demographic variables did not show differences in gender and age in the evaluation of health as a reason for visiting. As for other reasons, it was noted that the men valued more the leisure aspects, and differences were observed that did not reach statistical significance as a greater appreciation of the “plant-picking” reason in the female group. In regards to age, there were differences in the “enjoy” dimension, with a higher evaluation in those over 65 years and lower in those less than 30 years On the other hand, collection is a more frequent motivation in individuals older than 65 years (Table 1).

The relationships between the reasons and the beliefs were also studied, without there being any differences in the beliefs of the patients that go to Montseny NP-BR for health reasons. In regards to other reasons, those that went for “enjoyment” reasons were less likely to believe that nature benefits them.

### 3.4. Health-Related Activities in Montseny NP-BR

Exercise and collecting of medicinal plants were recorded as the main health-related activities that were carried out in the park.

#### 3.4.1. Exercise

The performing of exercise was mentioned by 82% of those surveyed, with a mean of 5 h weekly. More than half (58%) referred to the natural environment as the place that they usually performed exercise, with 29% in the urban nucleus, and 13% in sport installations. A total of 40% performed exercise exclusively in the natural environment. On being specifically asked about exercise in the Montseny NP-BR: 65% responded that they performed it between “1 and 3 times per week”, 24.8% recorded “almost never”, and 9% did not answer. In regards to the reason for performing exercise in the Montseny NP-BR, in 65% of cases, it was because it was considered healthier, and in 30% due to the evaluation of the environment. With a clear preference compared to the rest, the most common physical activity is “to walk” (79.2%), and in second place, “to ride a bicycle” (14.3%). In 56% of cases it was performed accompanied by another person, and 17% in a group. In 27% of those surveyed, it was an individual activity. The analysis by demographic variables showed that the patients over 50 years, evaluated health more as a reason for carrying out exercise in the natural environment. No differences were found in regards to gender (Table 1).

In regards to exercise and its frequency, it was noted that a greater number of patients that performed exercise among those that visited the Montseny NP-BR more often (more than once a week). On the other hand, those that frequent the park more often, evaluated the “pleasure” more as a reason for performing exercise in the natural environment.

The analysis of exercise in relation to the reasons for visiting the park showed that patients that go to the Montseny NP-BR for health reasons, perform more exercise. It should be pointed out that of the 66 patients that visited for health reasons, 63 performed exercise. These patients evaluated health in particular as a reason for practicing exercise in the park and, in second place, the environment. The individuals whose motivation for visiting the park was “to enjoy”, practiced exercise for less hours a week.

#### 3.4.2. Plant-Picking

**Figure 3:**
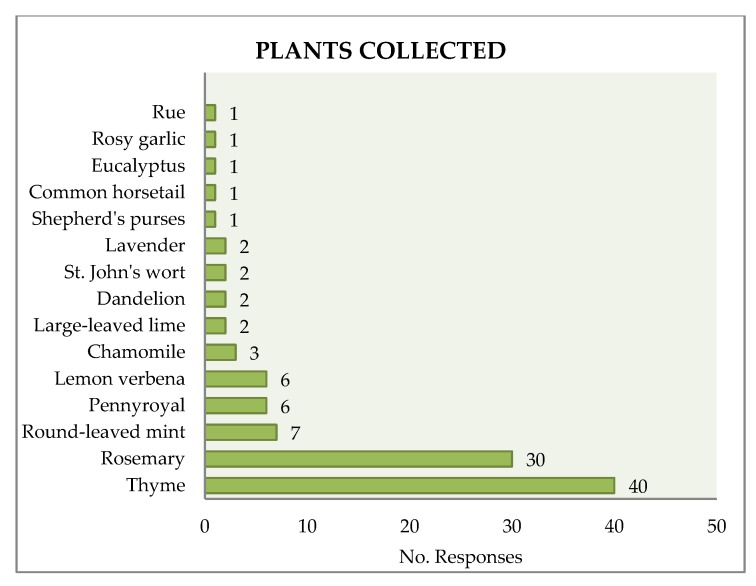
Species of plants collected.

More than one-third (39.6%) of those surveyed referred to collecting plants for medicinal uses. The most common species, clearly highlighted, were thyme (40 responses), rosemary (30) and to a lesser extent, mint (7), pennyroyal (6), and Maria Luisa (6) (Figure 3). Almost half (46%) of those surveyed collected mushrooms (16% habitually, and 30% occasionally). In the analysis by demographic variables, differences were observed that did not reach statistical significance in regards to a greater plant collection by women (Table 1). Furthermore, it was noted that women over 65 years had a higher collection activity (Table 1). No relationships were observed between beliefs, reasons for visiting the park, and plant collection.

### 3.5. Beliefs, Motivations, and Activities in Relation to the Diseases Suffered

The analysis of the data has been performed by using the most common disease groups: hypertension, obesity, diabetes, heart diseases, respiratory diseases, allergies, osteoarthritis, and anxiety/depression. In Table 2 and Table 3, the incidence of particular beliefs, resasons for visitting the park and the practice of health related activities is shown for the whole cohort of patients and for patients suffering from each disease group. In terms of beliefs, no differences were observed associated with the diseases when considering the benefit of nature for health. As for the reasons why it is considered beneficial, it was observed that those that suffered from allergies appreciated more the dimension associated with “pure air”. “Relaxation” is less valued by hypertensive and diabetic patients. Those with obesity also valued “relaxation” less, and differences that did not reach statistical significance are observed as a higher evaluation of “exercise” as a reason why nature is beneficial in this group.

There were no differences associated with the diseases in regards to the consideration of “health” as a reason why nature is beneficial. The patients with hypertension, diabetes, and osteoarthritis, valued “to enjoy” and “to collect” less, although it was more valued by the hypertensive patients. No differences were seen between those that suffered from heart diseases, respiratory diseases, anxiety and depression.

In regards to the reasons for visiting Montseny NP-BR, the patients with allergies and those with hypertension, valued the “leisure” reason more. In hypertensive patients, differences were observed that did not reach statistical significance as a higher evaluation of “collection”. On the other hand, this group, as well as the patients with diabetes, valued “to enjoy” less. No differences were seen between those that suffered from heart diseases, respiratory diseases, obesity, and anxiety and depression.

In regards to the activities that were carried out in Montseny NP-BR, no differences were observed in terms of frequency. Furthermore, no differences are observed in regards to the number of patients who performed exercise, although there was in the hours per week, with evidence that those who suffered from heart diseases performed more hours of exercise. Finally, no differences were found in the “collection” activity either (Table 2 and Table 3). 

## 4. Discussion

In regards to beliefs on the benefits of nature, a large majority (91%) of the patients surveyed considered that contact with nature is beneficial for health. Some of the reasons why it is considered beneficial, including the most valued responses overall, were those related the “pure air” dimension, recorded by approximately 1 in every 3 cases. This important valuation of pure air as a mechanism by which nature provides benefits for health, coincides with the scientific evidence that shows the important role of the natural environment in the purification of the air through the objectively recognized reduction in contaminating atmospheric [87,88,94]. Likewise, the benefits that this purifying function has on health has also been demonstrated, and in particular, on the mortality associated with respiratory diseases [55]. In regards to this latter point, a higher evaluation of the pure air would have been expected from the group of patients with asthma or chronic obstructive pulmonary disease (COPD), a fact that has not been mentioned in the study, although those that suffered from allergies appreciated it more.

One point to highlight is the importance given to the aspects associated with the reduction in stress, supported by the fact that tranquility may be one of the main mechanisms why those that consider that nature is beneficial and that, likewise, may be one of the main reasons for visiting the Montseny NP-BR.

These results agree with similar studies performed on a general population, both in PNAs in Spain [95,96,97,98], as well in other European countries [99,100,101], in which it is shown that the aspects associated with the reduction in stress is one of the most important reasons for visiting PNAs. These data corroborate the importance of the restorative power against stress as one of the most positive evaluations attributed to the natural environment [102,103], and one of the most important mediators through which the benefits of nature are produced [50,57,59].

On the other hand, the fact that it was most valued by women, agrees with the findings by other studies that show that the mediators through which nature is beneficial (exercise, relaxation, etc.), have a different weight depending on gender and age [17,104]. In terms of the diseases, despite the fact that tranquility has an important weight overall, there is no higher evaluation by those individuals with anxiety and depression disorders.

One point that should be highlighted is the low evaluation of exercise among the reasons why nature is beneficial. This information contrasts with the results of many studied that show physical activity as one of the most important mediators of the benefits of the natural environment for health. This low evaluation could be justified by the fact that, on being a free response question, it is likely that exercise is included in the health response. On the other hand, this would corroborate the importance of the study of the beliefs in health to contribute to developing the planning of recourses that the natural environment can offer in relation to health.

The most important reasons for visiting the park are found in the dimensions related to “enjoy” (44.8%) and “tranquility” (40.4%). The importance of “enjoy” as a reason for visiting a PNA varies other studies [96,105], probably due to the fact that is not a very specific term, and with wide significance.

An important valuation is observed on the aspects associated with “health” as a reason for visiting Montseny PN-RB; in the general valuation it is recorded in 1 in every 4 individuals and, when, questioned directly, 1 in 2 acknowledge that they visit the park in order to benefit their health. No differences were found in gender and age, despite the fact that other studies show a higher valuation of this reason in women [106].

In studies conducted on the general population regarding PNA [95,96], health is valued after enjoy, tranquility, and contact with nature. Among the studies in which health is most valued as a reason for visiting, the one conducted in Catalonia is highlighted [107], in which health was the most important reason for visiting four ANPs of Barcelona, such as the study conducted in Poland, in the Wigierski National Park [106].

In another two studies, carried out in the PNAs of Canada [19] and in the Parque Natural Baixa Limia-Serra do Xurés in Spain [97], health was the second most important reason after the tranquility. In this last case, the important motivation of health is justified by the presence of thermal waters. This would corroborate the observation that the evaluations and activities performed in the natural spaces are also influenced by their environmental surroundings [108]. On the other hand, this evaluation of health agrees with the results of studies that investigate the perceived benefits after visiting PNA, in that physical well-being has a secondary evaluation, behind the psychological and social benefits [19,90,98,109].

In the case of the Montseny NP-BR, the important evaluation of health could be mediated by the fact that the study has been carried out on a group of individuals with a higher rate of diseases than the general population, in those that it would be expected that the aspects related to lifestyle and health would have greater relevance [110]. These results acknowledge the importance of promoting health resources linked to the natural environment of populations with specific needs, as in the case of patients with certain diseases. On the other hand, the Montseny NP-BR has a historical role as a therapeutic landscape, a fact that could also justify that the individuals that visit it have this reason more in mind [111]. In regards to this point, few studies have currently approached the topic of the reasons and perceived benefits in relation to the visit to these natural spaces in those in which historically, health has increasingly gained importance.

In regards to activities associated with health in the park, there is evidence of a relationship between exercise, health as a reason for visiting, and the frequency of visiting the Montseny NP-BR. Likewise, it is noted that an elevated number of patients perform exercise in the park environment.

Significant levels of exercise are recorded (82%), above that of the mean for the general population of Catalonia (74.2%) [112]. Furthermore, the percentages are higher than those recorded in Europe (29.9%) [113], and in the USA (51.7%) [114], and superior to the data acknowledged by the WHO that warns that 1 in every 4 individuals have insufficient levels of physical activity [115]. The verification of exercise levels in a patient population group is especially significant given the important role of physical activity in the therapeutic management of a large number of diseases. One point to underline is the significant level of physical exercise performed by the patient group with cardiovascular diseases, greater than that referred to in different patient registers [116,117]. It is an important fact, given the crucial role of physical activity in the prevention and treatment of these diseases [118].

Despite there being no data that could establish that the proximity of the Montseny NP-BR could be a reason for this level of exercise, although there is a correlation between the frequency of visiting the park and exercise, as such that the patients that perform exercise are also those that visit the park more often. A considerable frequency of visiting the Montseny NP-BR is also recorded (1 in every 3 visit it more than once a week). The characteristics of the environment in which it resides play and important role in the physical activity [119], as such that natural landscapes are preferred for perform exercise [120]. On the other hand, several studies demonstrate that people that live in areas near natural spaces carry out more physical activity [21,49,57], especially if they are places evaluated as having an elevated recreational value [121]. Furthermore, this fact should be found in the line of investigations that show that individuals who reside in rural areas perform more physical activity than those that live in urban areas [122,123,124].

Another relevant piece of data is the significant number of patients that perform exercise in the Montseny NP-BR (65%), and the evidence that health may be the main reason, higher than the evaluation of the aesthetic environment. On the other hand, in different series on the activities that are conducted on visitors to PNAs, physical activity occupies a prominent position [95,98,125]. These facts should corroborate the facilitator role of physical activity as one of the mediators through which the benefits of the natural environment are produced [79], as well as the contribution of the PNAs to human health.

The majority of studies that analyze the environment in which physical activity is carried out are focused on urban areas, and there are few specific registers in populations near to PNAs. In the review of the literature performed by [126], different studies showed that, in the urban environment, approximately 20–30% of the individuals performed exercise in parks [127], with streets and shopping centers being the most common places for carrying out physical activity [128,129]. Furthermore, the study conducted by [130] showed that, 38% of the individuals from urban areas exercised in parks compared to 22% in suburban areas, and 17% in the rural environment.

One notable piece of date in regards to other studies is the important activity of plant collecting mentioned in the Montseny NP-BR: 39.6% of those surveyed mentioned collecting plants for medicinal uses, and 46% collected mushrooms. This relevance is also reflected in the fact that it is a common reason (28%) for visiting the park. This plant picking activity is probably associated with the important tradition in Catalonia and in the Iberian Peninsula that, despite it having decreased overall, it currently continues to be an activity most associated with recreational values [131], particularly in rural areas [132]. This could also justify the fact that this activity was more common in this study among older patients. Women also showed a significantly higher plant collecting activity, a fact that is also associated with other observations [133].

Despite the fact that the questionnaire did not ask for the reason for plant collecting, the context leads to considering that is not an economic activity but it is more for leisure reasons and personal consumption. On the other hand, although it was specifically asked about the collecting of medicinal plants, the majority of the responses recorded (thyme and rosemary) are species that, as well as their therapeutic properties, have a wide culinary use in the area. In regards to the diseases, despite there being no significant differences associated with plant collection, a higher evaluation of this activity is recorded among the hypertensive patients, both as a reason for visiting nature, as well as a reason for which they believed that it was beneficial. This fact could be justified by the diuretic properties of several plant species, valued in the treatment of hypertension. Finally, it is pointed out that the topic of plant collecting is barely mentioned in the studies on activities carried out by visitors to PNAs. This is probably due to the fact that many protected natural areas restrict or prohibit harvesting although legislation on this subject is irregular. On the other hand, in the ANP there are plants for medicinal and culinary use that may be useful, although their collection may contradict conservation programs. The potential to link collection with conservation in protected areas has been considered in Southeast Europe, which has traditionally been one of Europe’s most important source regions for medicinal and aromatic plants [134].

As limitations of the study, to point out that the results are not compared with different populations, such as healthy individuals, or visitors that do not live in the proximity of the park.

As future lines of research in relation to protected PNAs and health, it is considered that, in addition to promoting the preventive role in healthy individuals, they could be a resource for population groups with particular diseases, with the possibility of implementing specific measures like respiratory physiotherapy, or rehabilitation programs after a cardiovascular event. In this sense, it would be interesting to widen the study of potential benefits that the natural environment can exert on individuals with health problems, like, for example, studying the effect that contact with nature may have on the stress parameters of individuals with anxiety, or changes in the respiratory functions tests, including those with asthma.

## 5. Conclusions

An important evaluation of the natural environment as beneficial for the health of patients is presented. The close relationship between contact with nature and physical exercise is also demonstrated, as well as a significant health-associated plant collecting activity.

The study ratifies the significant role of Montseny NP-BR as a health resource for patients with different diseases that reside in its proximity. On the other hand, it corroborates the beneficial effects that the PNAs provide in human health and well-being and contributes to the study of their role in relation to health, an emerging health topic, scarcely treated in the literature. Likewise, it confirms the role of the natural environment as a facilitator of healthy habits such as physical exercise, contributing to the growing evidence of the benefits that nature has for human health.

As a contribution, the study has a health-centered vision, focusing attention on a population of individuals with diseases in which the potential benefits that the natural environment has for their health, has been scarcely treated. Furthermore, up until now, the role that the PNAs may have in the health of those individual who live near them has not been approached. Another contribution is the analysis of beliefs in health as an element that, on providing information together with the opinions and need of the individuals, could contribute to managing the resources of the natural environment in public health programs. Finally, the study on the collecting of plants for medicinal purposes should be pointed out, which was scarcely approached as a reason for visiting, as well as an activity associated with health in the PNAs.

We consider this knowledge in the interest of promoting health values in relation to the PNAs, as the study of these areas, such as Montseny NP-BR, have the added value of their historic role as therapeutic landscapes.

The protected natural areas, with their contribution to the health and well-being of individuals, can be an important element to take into consideration in public health management programs.

## Figures and Tables

**Table 1:** Beliefs on the benefit of nature, reasons for visiting Montseny NP-BR and activities according to gender and age.

	Patients	Sex			Age				
	*n* = 250	Man *n* = 133	Women *n* = 115	*p*-Value	16–30 *n* = 7	31–50 *n* = 57	51–65 *n* = 68	>65 *n* = 116	*p*-Value
	*n* (%)	*n* (%)	*n* (%)		*n* (%)	*n* (%)	*n* (%)	*n* (%)	
**Reasons for Visiting Montseny NP-BR**									
Enjoy	112 (44.8)	54 (40.6)	58 (50.4)	0.155	5 (4.46)	34 (30.4)	26 (23.2)	47 (42.0)	0.028 **
Health	66 (26.4)	33 (24.8)	32 (27.8)	0.694	1 (1.52)	16 (24.2)	22 (33.3)	27 (40.9)	0.534
Tranquility	101 (40.4)	50 (37.6)	51 (44.3)	0.342	3 (3.00)	30 (30.0)	29 (29.0)	38 (38.0)	0.082*
Leisure	42 (16.8)	29 (21,8)	13 (11.3)	0.042 **	0 (0.00)	6 (14.6)	9 (22.0)	26 (63.4)	0.123
Plant-picking	70 (28.0)	45 (33.8)	53 (46.1)	0.066 *	3 (4.35)	9 (13.0)	22 (31.9)	35 (50.7)	0.088 *
**Benefits of Nature in Relation to Health**									
Pure air	68 (27.2)	32 (24.1)	36 (31.3)	0.257	1 (14.3)	20 (35.1)	16 (23.5)	30 (25.9)	0.451
Tranquility	44 (17.6)	18 (13,5)	25 (21,7)	0.125	1 (14.3)	12 (21.1)	17 (25.0)	14 (12.1)	0.117
Health	25 (10.0)	14 (10.5)	11 (9.57)	0.969	1 (14.3)	5 (8.77)	10 (14.7)	9 (7.76)	0.365
Exercise	12 (4.80)	8 (6.20)	4 (3.48)	0.528	0 (0.00)	3 (5.26)	4 (5.88)	5 (4.31)	0.905
Spiritual	2 (0.80)	1 (0.75)	1 (0.87)	1.000	0 (0.00)	1 (1.75)	0 (0.00)	1 (0.86)	0.525
**Health-Related Activities**									
Exercise	204 (81.9)	107 (81.1)	95 (82.6)	0.881	5 (71.4)	48 (84.2)	55 (80.9)	94 (81.7)	0.816
Plant-picking	99 (39.6)	45 (33.8)	53 (46.1)	0.066 *	3 (4.35)	9 (13.0)	22 (31.9)	35 (50.7)	0.088 *

* *p* < 0.05; ** *p* < 0.01. Note: the same subject may indicate several reasons. The percentages indicate the incidence of the criterion in the subpopulation of the corresponding column. The *p*-values for χ^2^ tests, compare differences in the percentages of each criterion between the subpopulations of the corresponding columns.

**Table 2:** Beliefs on the benefits of nature, reasons for visiting Montseny PN-RB, and activities depending on the diseases.

	Patients *n* = 250	Diabetes *n* = 52		Obesity *n* = 71		Heart Dis. *n* = 107		Hypertension *n* = 147	
	*n* (%)	*n* (%)	*p*-value	*n* (%)	*p*-value	*n* (%)	*p*-value	*n* (%)	*p*-value
**Reasons for Visiting Montseny NP-BR**									
Enjoy	112 (44.8)	16 (30.8)	0.031 **	28 (39.4)	0.374	42 (39.3)	0.162	58 (39.5)	0.041 **
Health	66 (26.4)	14 (26.9)	1.000	18 (25.4)	0.919	28 (26.2)	1.000	39 (26.5)	1.000
Tranquility	101 (40.4)	19 (36.5)	0.622	33 (46.5)	0.290	46 (43.0)	0.554	58 (39.5)	0.838
Leisure	42 (16.8)	12 (23.1)	0.192	9 (12,7)	0.353	20 (18.7)	0.602	32 (21.8)	0.023 **
Plant-picking	70 (28.0)	19 (36.5)	0.167	21 (29.6)	0.796	32 (29.9)	0.661	48 (32.7)	0.057
**Benefits of Nature in Relation to Health**									
Pure air	68 (27.2)	13 (25.0)	0.889	19 (26.8)	1.000	24 (22.4)	0.168	37 (25.2)	0.635
Tranquility	44 (17.6)	3 (5.77)	0.019 **	6 (8.45)	0.026 **	16 (15.0)	0.434	18 (12.2)	0.010 **
Health	25 (10.0)	7 (13.5)	0.522	8 (11.3)	0.862	12 (11.2)	0.733	17 (11.6)	0.470
Exercise	12 (4.80)	2 (3.85)	1.000	6 (8.45)	0.106	7 (6.54)	0.415	9 (6.12)	0.369
Spiritual	2 (0.80)	0 (0.00)	1.000	1 (1.41)	0.490	1 (0.93)	1.000	0 (0.00)	0.165
**Health-Related Activities**									
Exercise	204 (81.9)	38 (74.5)	0.166	55 (77.5)	0.340	85 (80.2)	0.655	118 (80.3)	0.433
Plant-picking	99 (39.6)	19 (36.5)	0.768	26 (36.6)	0.620	46 (43.0)	0.414	64 (43.5)	0.153

* *p* < 0.05; ** *p* < 0.01. Note: a single patient may appear under different diseases and may indicate several reasons. The percentages indicate the incidence of the criterion in the sub-population of the corresponding column. The *p*-values for individual χ^2^ tests, correspond to the comparison between the patients with the diseases of the particular group compared to patients that did not suffer from them (although they could suffer from others).

**Table 3:** Beliefs on the benefits of nature, reasons for visiting Montseny PN-RB, and activities depending on the diseases.

	Patients *n* = 250	Allergies *n* = 47		Respiratory Dis. *n* = 51		Anx./Depress. *n* = 78		Osteoarthritis *n* = 79	
		*n* (%)	*p*-value	*n* (%)	*p*-value	*n* (%)	*p*-value	*n* (%)	*p*-value
**Reasons for Visiting Montseny NP-BR**									
Enjoy	112 (44.8)	20 (42.6)	0.856	42 (82.4)	1.000	34 (46.6)	0.903	26 (32.9)	0.015 **
Health	66 (26.4)	15 (31.9)	0.442	16 (31.4)	0.468	25 (32.1)	0.226	24 (30.4)	0.415
Tranquility	101 (40.4)	22 (46.8)	0.407	24 (47.1)	0.354	37 (47.4)	0.165	31 (39.2)	0.908
Leisure	42 (16.8)	14 (29.8)	0.015**	9 (17.6)	1.000	8 (10.3)	0.093 *	16 (20.3)	0.418
Plant-picking	70 (28.0)	11 (23.4)	0.550	13 (25.5)	0.785	24 (30.8)	0.614	20 (25.3)	0.624
**Benefits of Nature in Relation to Health**									
Pure air	68 (27.2)	19 (40.4)	0.038 **	16 (31.4)	0.566	26 (3.3)	0.189	15 (19.0)	0.067 *
Tranquility	44 (17.6)	7 (14.9)	0.743	7 (13.7)	0.543	15 19.2)	0.782	13 (16.5)	0.885
Health	25 (10.0)	3 (6.38)	0.558	6 (11.8)	0.834	9 (11.5)	0.750	8 (10.1)	1.000
Exercise	12 (4.80)	3 (6.38)	0.558	2 (3.92)	1.000	1 (1.28)	0.111	4 (5.06)	1.000
Spiritual	2 (0.80)	1 (2.13)	0.341	0 (0.00)	1.000	1 (1.28)	0.528	1 (1.27)	0.533
**Health-Related Activities**									
Exercise	204 (81.9)	39 (83.0)	1.000	42 (82.4)	1.000	65 (83.3)	0.832	64 (81.0)	0.937
Plant-picking	99 (39.6)	22 (46.8)	0.339 **	20 (39.2)	1.000	30 (38.5)	0.914	28 (35.4)	0.439

* *p* < 0.05; ** *p* < 0.01. Note: a single patient may appear under different diseases and may indicate several reasons. The percentages indicate the incidence of the criterion in the sub-population of the corresponding column. The *p*-values for individual χ^2^ tests, correspond to the comparison between the patients with the diseases of the particular group compared to patients that did not suffer from them (although they could suffer from others).
